# Enhanced balance associated with coordination training with stochastic resonance stimulation in subjects with functional ankle instability: an experimental trial

**DOI:** 10.1186/1743-0003-4-47

**Published:** 2007-12-17

**Authors:** Scott E Ross, Brent L Arnold, J Troy Blackburn, Cathleen N Brown, Kevin M Guskiewicz

**Affiliations:** 1 Department of Health and Human Performance, Virginia Commonwealth University, Richmond, VA, USA; 2 Department of Exercise and Sport Science, University of North Carolina at Chapel Hill, Chapel Hill, NC, USA; 3 Department of Kinesiology, The University of Georgia, Athens, GA, USA

## Abstract

**Background:**

Ankle sprains are common injuries that often lead to functional ankle instability (FAI), which is a pathology defined by sensations of instability at the ankle and recurrent ankle sprain injury. Poor postural stability has been associated with FAI, and sports medicine clinicians rehabilitate balance deficits to prevent ankle sprains. Subsensory electrical noise known as stochastic resonance (SR) stimulation has been used in conjunction with coordination training to improve dynamic postural instabilities associated with FAI. However, unlike static postural deficits, dynamic impairments have not been indicative of ankle sprain injury. Therefore, the purpose of this study was to examine the effects of coordination training with or without SR stimulation on static postural stability. Improving postural instabilities associated with FAI has implications for increasing ankle joint stability and decreasing recurrent ankle sprains.

**Methods:**

This study was conducted in a research laboratory. Thirty subjects with FAI were randomly assigned to either a: 1) conventional coordination training group (CCT); 2) SR stimulation coordination training group (SCT); or 3) control group. Training groups performed coordination exercises for six weeks. The SCT group received SR stimulation during training, while the CCT group only performed coordination training. Single leg postural stability was measured after the completion of balance training. Static postural stability was quantified on a force plate using anterior/posterior (A/P) and medial/lateral (M/L) center-of-pressure velocity (COPvel), M/L COP standard deviation (COPsd), M/L COP maximum excursion (COPmax), and COP area (COParea).

**Results:**

Treatment effects comparing posttest to pretest COP measures were highest for the SCT group. At posttest, the SCT group had reduced A/P COPvel (2.3 ± 0.4 cm/s vs. 2.7 ± 0.6 cm/s), M/L COPvel (2.6 ± 0.5 cm/s vs. 2.9 ± 0.5 cm/s), M/L COPsd (0.63 ± 0.12 cm vs. 0.73 ± 0.11 cm), M/L COPmax (1.76 ± 0.25 cm vs. 1.98 ± 0.25 cm), and COParea (0.13 ± 0.03 cm^2 ^vs. 0.16 ± 0.04 cm^2^) than the pooled means of the CCT and control groups (P < 0.05).

**Conclusion:**

Reduced values in COP measures indicated postural stability improvements. Thus, six weeks of coordination training with SR stimulation enhanced postural stability. Future research should examine the use of SR stimulation for decreasing recurrent ankle sprain injury in physically active individuals with FAI.

## Background

Ankle sprains are common sports injuries that occur frequently in the physically active [1,2]. Residual symptoms can exist following ankle sprains, and often lead to a pathology known as functional ankle instability (FAI) [3]. Physically active individuals with FAI report feelings of ankle instability and recurrent ankle sprains with activity [3,4]. Interestingly, the underlying cause of FAI is unclear even though this pathology is prevalent in individuals with a history of ankle sprain injury. Researchers have suggested that FAI develops from sensorimotor dysfunctions, strength deficits, mechanical instability, or a combination of the aforementioned factors [5-8].

The sensorimotor system is responsible for maintaining functional joint stability by integrating afferent and efferent signals with central information to activate dynamic restraints surrounding joints [9]. Sensorimotor system impairments associated with FAI have been demonstrated while balancing on a single leg [5-7,10-12]. Poor sensory integration of afferent and efferent signals might impair postural stability by disrupting reflexive and feedforward neuromuscular responses, resulting in excessive sway during single leg stance in individuals with FAI [12,13].

Postural stability impairments are predictors of ankle sprain injury [14-16] and have been related to FAI [5-7,10-12]. Sports medicine clinicians and researchers have used coordination training as a therapy to rehabilitate FAI, as well as to improve postural stability deficits associated with FAI [17-22]. Coordination training is thought to enhance sensorimotor function and, thereby, improve postural stability [17-22]. Furthermore, enhanced sensorimotor function has been associated with improvements in ankle stability, [19,20,22,23] and has reduced the incidence of ankle sprain injury in individuals with FAI [1,20,23,24]. However, a number of physically active individuals who have participated in coordination training or other ankle rehabilitation protocols still sustain ankle sprain injuries [1,20,23,24]. The stimulus from ankle rehabilitation might not be strong enough to enhance the sensorimotor system in individuals with FAI who do not achieve the full prophylactic effects associated with rehabilitation [2,25,26]. Therapy providing a greater treatment effect than coordination training alone, for example, might have implications for preventing ankle sprain injury.

Stochastic resonance (SR) stimulation in the form of subsensory electrical noise or mechanical noise applied to the skin might be a therapy used to improve postural stability. Stochastic resonance stimulation introduces low levels of noise into the nervous system to enhance the detection of sensorimotor signals related to postural control [27-30]. In other words, SR stimulation in the form of random subsensory electrical noise causes sub-threshold sensorimotor signals to exceed threshold, allowing weak sensorimotor signals related to joint motion to become detectable [31]. Evidence also indicates that SR stimulation enhances monosynaptic reflex responses generated by muscle spindles [32]. Thus, this information indicates that SR stimulation enhances the sensitivity of sensorimotor input and affects central nervous system output. Stochastic resonance stimulation therapy has been useful for improving postural stability in healthy young and elderly individuals when compared to postural stability tests without stimulation [27-30].

Recently, coordination training with SR stimulation has been reported to improve dynamic postural stability earlier and to a greater extent than coordination training without SR stimulation [22]. The effect of coordination training with SR stimulation on static postural stability also should be examined since single leg postural stability deficits have been associated with FAI [5-7,10-12] and have predicted ankle sprain injury in physically active individuals [14-16]. Therefore, the purpose of this study was to examine the effects of six weeks of coordination training with or without SR stimulation on static postural stability of subjects with FAI.

## Methods

### Subjects

Sixteen females and fourteen males (177 ± 10 cm, 76 ± 16 kg, 21 ± 2 years) with FAI from a larger study served as subjects for this study [22]. All subjects received a test protocol orientation prior to their participation in this study. Subjects read and signed a consent form approved by The Committee for the Protection of the Rights of Human Subjects.

All subjects reported a history of a severe ankle sprain injury that required immobilization, as well as a minimum of two ankle sprains and two "giving way" sensations within the year prior to data collection. The majority of our subjects had mechanical instability (67% with anterior drawer laxity and 76% with talar tilt laxity). Potential subjects with FAI were excluded if they had an ankle sprain injury within six weeks prior to their participation or participated in an ankle rehabilitation program six weeks prior to this study.

### Coordination training

Subjects were randomly assigned to either a: 1) conventional coordination training group (CCT) composed of 10 subjects; 2) SR stimulation coordination training group (SCT) composed of 10 subjects; or 3) control group composed of 10 subjects. The training groups performed coordination training 5 times per week for six weeks on their leg with FAI (test leg). Single leg coordination exercises performed in this investigation included balance on foam (3 sets × 30 s), circular motion on a wobble board (2 sets × 60 s), and resistance band kicks (3 sets × 120 repetitions). Detailed descriptions of these exercises are published in a previous report [22].

Subjects in both training groups were shoeless while training and wore SR stimulator units (Afferent Corp., Providence, RI) with surface electrode (2 × 2 cm) self-adhesive gel pads (Model Platinum 896230, Axelgaard Mfg. Co., Ltd., Fallbrook, CA) on the skin over the muscle bellies of the lateral soleus, peroneus longus, tibialis anterior, anterior talofibular ligament, and deltoid ligament of the test leg. Both groups were required to wear SR stimulator units during training to reduce the likelihood of a "placebo/sham" effect. Subjects were blinded to their training group, as the stimulation delivered to the SCT group was subsensory (Gaussian white, zero mean, sd = 0.05 mA, band-pass filtered below 1000 Hz). No stimulation was applied to the CCT group. The control group did not participate in coordination training.

### Single leg stance test

Subjects wore shoes during the single leg stance test. The SR stimulators were not worn by subjects during this test. Subjects placed their foot with FAI (i.e., the test leg) in a comfortable position while standing in the center of a force plate. Subjects kept their eyes open, hands on their hips, and their non-weight bearing limb in a slightly flexed position. Subjects were instructed to remain as motionless as possible for 20 s. Subjects performed 1 practice trial and then performed 3 test trials. Trials were discarded and repeated if subjects touched their non-weight bearing leg to the floor.

### Data collection

A force plate (Bertec Corp., Columbus, Ohio) collected analog data at a sampling rate of 180 Hz [10]. Analog signals were amplified by a factor of 2 and passed through a BNC adapter chassis (National Instruments model # PCI-MIO-16E-1) that was interfaced with a 12 bit analog-to-digital converter within a personal computer. MotionSoft Balance Assessment computer software package version 2.0 (MotionSoft Inc., Chapel Hill, NC) converted digital data to ground reaction forces, moments, and center-of-pressure. Data were then filtered with a 2^nd ^order recursive low-pass Butterworth digital filter with an estimated optimum cutoff frequency of 12.53 Hz [10].

Table 1 presents the five center-of-pressure (COP) measures calculated to assess postural stability in this study. The five COP measures used in this study were: anterior/posterior (A/P) sway velocity (A/P COPvel), medial/lateral (M/L) sway velocity (M/L COPvel), M/L standard deviation (M/L COPsd), M/L maximum excursion (M/L COPmax), and area (COParea). The COPvel, M/L COPsd, M/L COPmax, and COParea measures have detected treatment effects associated with SR stimulation and coordination training in subjects with FAI [20,21,27]. Additionally, COPvel and COPsd measures have been indicative of ankle sprain injury [15,16]. Reduced variations in M/L COPsd, shorter excursions of M/L COPmax, less area in COParea, and slower velocities in COPvel are indicative of improved postural stability.

**Table 1:** Center-of-Pressure Calculations.

COPvel: The mean value of the instantaneous velocity of the COP in a given direction during a given time period	$\begin{array}{l} \text{A/P COPvel}=\frac{\sum_{\text{t}=1}^{\text{T}}\left|\frac{{\text{x}}_{\text{cop,t}}-{\text{x}}_{\text{cop,t}-1}}{\Delta \text{t}}\right|}{\text{T}-1}\\ \text{M/L COPvel}=\frac{\sum_{\text{t}=1}^{\text{T}}\left|\frac{{\text{y}}_{\text{cop,t}}-{\text{y}}_{\text{cop,t}-1}}{\Delta \text{t}}\right|}{\text{T}-1} \end{array}$
M/L COPsd: Overall standard deviation of sway in the M/L direction in a given time period for a given number of trials	$\begin{array}{c} \text{M/L COPsd}=\sqrt{\frac{\sum_{\text{n}=1}^{\text{N}}\sum_{\text{t}=0}^{\text{T}}{\text{Sway}}_{\text{M}/\text{L,t,n}}^{2}-\frac{{\left(\sum_{\text{n}=1}^{\text{N}}\sum_{\text{t}=0}^{\text{T}}{\text{Sway}}_{\text{M/L,t,n}}\right)}^{2}}{\text{N}(\text{T}-1)}}{\text{NT}-1}}\\ {}*{\text{Sway}}_{\text{M/L}}=\frac{\sum_{\text{t}=0}^{\text{T}}\left|{\text{COP}}_{\text{M/L,t}}-{\text{COP}}_{\text{M/L,mean}}\right|}{\text{T}} \end{array}$
COParea: An area defined by the maximum (max) anterior (ant), posterior (post), medial (med), and lateral (lat) sways during a given time period.	$\begin{array}{c} \text{COParea}=\frac{\left({\text{Sway}}_{\text{max,ant}}+{\text{Sway}}_{\text{max,post}})\times ({\text{Sway}}_{\text{max,med}}+{\text{Sway}}_{\text{max,lat}}\right)}{\text{T}}\\ {}*{\text{Sway}}_{\text{max,direction}}=\frac{\sum_{\text{t}=0}^{\text{T}}\left|{\text{COP}}_{\text{direction,t}}-{\text{COP}}_{\text{direction,mean}}\right|}{\text{T}} \end{array}$
M/L COPmax: Maximum distance between the instantaneous COP position and the average COP position during a given time period.	$\text{M/L COPmax}=\frac{\sum_{\text{t}=0}^{\text{T}}\left|{\text{COP}}_{\text{max,M/L}}-{\text{COP}}_{\text{M/L,mean}}\right|}{\text{T}}$

Calculations for the following postural stability measures are presented in Table 1: Anterior/Posterior Center-of-Pressure Velocity (A/P COPvel); Medial/Lateral Center-of-Pressure Velocity (M/L COPvel); Medial/Lateral Center-of-Pressure Standard Deviation (M/L COPsd); Center-of-Pressure Area (COParea); and Medial/Lateral Center-of-Pressure Maximum Excursion (M/L COPmax). t = A given time point; T = Number of data points per trial; N = Number of trials

### Statistical analysis

The mean of 3 trials for single leg stance testing for pre- and post-tests were used for data analysis. Separate planned orthogonal contrasts were used to analyze differences between group means for each dependent measure at pre- and post-tests. The orthogonal contrasts for the pretest data examined differences between the control, CCT, and SCT groups using two-tailed t-tests. Two-tailed t-tests were used to detect decreased or increased balance differences between groups. Orthogonal contrasts for posttest data examined differences between the control, CCT, and SCT groups using one-tailed t-tests. One-tailed t-tests were used to detect balance improvements in groups, as we did not expect balance to worsen after training or for the control group. The first orthogonal contrast for the dependent measures examined differences between the control and CCT groups. The second orthogonal contrast for the dependent measures examined differences between the SCT group and the pooled mean of the control and CCT groups. Cohen's [33] effect size (ES) *d *examined our treatment effect by comparing differences between the pooled pretest mean of all groups and each groups' respective posttest data. SPSS version 13.0 (SPSS Inc., Chicago, IL) was used for statistical analysis. Alpha was set a priori at P < 0.05 to indicate statistical significance.

## Results

**Figure 1:**
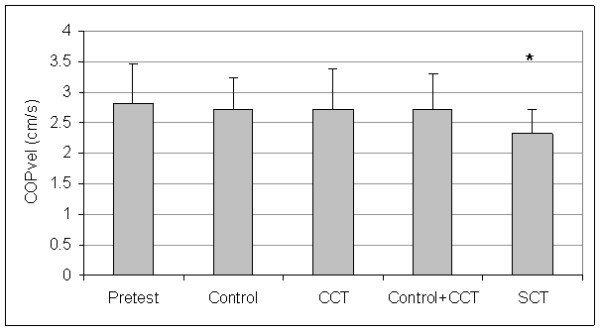
**Means And Standard Deviations Of Anterior/Posterior Center-Of-Pressure Velocity (A/P COPvel)**. *The stochastic resonance stimulation coordination training (SCT) group had slower posttest A/P COPvel than the posttest pooled mean of the control and conventional coordination training (CCT) groups. Pretest = A/P COPvel pooled pretest means of all groups.

**Figure 2:**
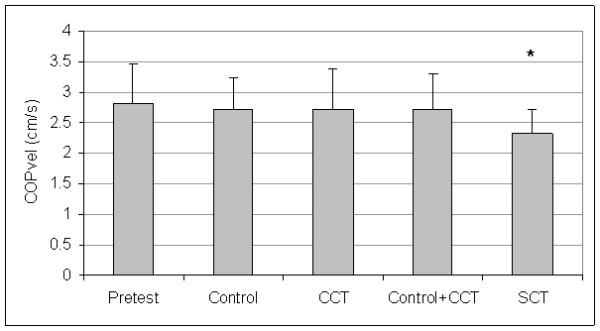
**Means And Standard Deviations Of Medial/Lateral Center-Of-Pressure Velocity (M/L COPvel)**. *The stochastic resonance stimulation coordination training (SCT) group had slower posttest M/L COPvel than the posttest pooled mean of the control and conventional coordination training (CCT) groups. Pretest = M/L COPvel pooled pretest means of all groups.

**Figure 3:**
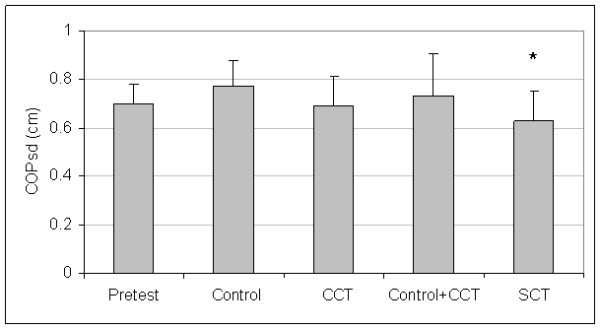
**Means And Standard Deviations Of Medial/Lateral Center-Of-Pressure Standard Deviation (M/L COPsd)**. *The stochastic resonance stimulation coordination training (SCT) group had reduced posttest M/L COPsd than the posttest pooled mean of the control and conventional coordination training (CCT) groups. Pretest = M/L COPsd pooled pretest means of all groups.

**Figure 4:**
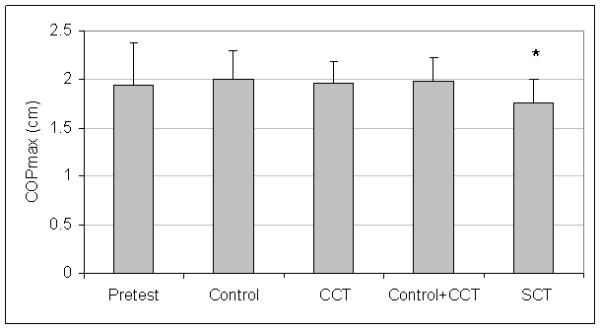
**Means And Standard Deviations Of Medial/Lateral Center-Of-Pressure Maximum Excursion (M/L COPmax)**. *The stochastic resonance stimulation coordination training (SCT) group had shorter posttest M/L COPmax than the posttest pooled mean of the control and conventional coordination training (CCT) groups. Pretest = M/L COPmax pooled pretest means of all groups.

**Figure 5:**
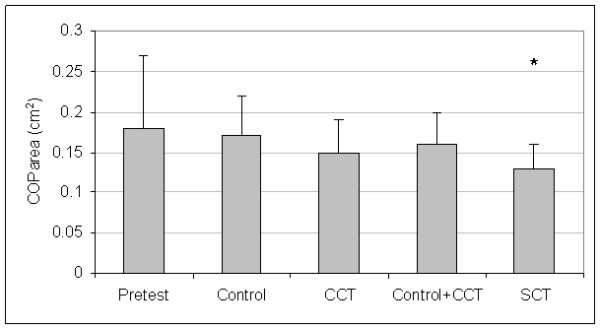
**Means And Standard Deviations Of Center-Of-Pressure Area (COParea)**. *The stochastic resonance stimulation coordination training (SCT) group had less posttest COParea than the posttest pooled mean of the control and conventional coordination training (CCT) groups. Pretest = COParea pooled pretest means of all groups.

Control and CCT group pretest means were not different for A/P COPvel (t_(27) _= 0.46, P = 0.652), M/L COPvel (t_(27) _= -0.27, P = 0.787), M/L COPsd (t_(27) _= -1.02, P = 0.319), M/L COPmax (t_(27) _= -0.84, P = 0.410), or COParea (t_(27) _= -1.02, P = 0.319). The SCT and pooled (control + CCT) pretest means were not different for A/P COPvel (t_(27) _= 0.53, P = 0.604), M/L COPvel (t_(27) _= 1.09, P = 0.287), M/L COPsd (t_(27) _= 1.16, P = 0.254), M/L COPmax (t_(27) _= 0.69, P = 0.499), or COParea (t_(27) _= 1.23, P = 0.229). Since group differences were not present at pretest, the pretest data for all groups were averaged to create pretest pooled means for each dependent measure. Figures 1, 2, 3, 4, and 5 present the pooled pretest means (standard deviations).

The control and CCT posttest mean comparisons were not different for A/P COPvel (t_(27) _= 0.01, P = 0.497), M/L COPvel (t_(27) _= -0.43, P = 0.334), M/L COPsd (t_(27) _= -1.54, P = 0.068), M/L COPmax (t_(27) _= -0.31, P = 0.382), or COParea (t_(27) _= -0.73, P = 0.236). However, the SCT group had reduced posttest means than pooled (control + CCT) posttest means for A/P COPvel (t_(27) _= 1.88, P = 0.036), M/L COPvel (t_(27) _= 1.71, P = 0.049), M/L COPsd (t_(27) _= -2.37, P = 0.013), M/L COPmax (t_(27) _= 2.29, P = 0.015), and COParea (t_(27) _= 1.79, P = 0.043). Figures 1, 2, 3, 4, and 5 present posttest means (standard deviations) for each group.

Table 2 presents the treatment effect associated with posttest improvements in postural stability compared to the pooled pretest means. In general, effect sizes for the control and CCT groups were low, indicating postural stability did not improve at posttest. In some cases, low negative and moderately negative effect sizes were found for control and CCT groups, indicating postural stability impairments at posttest. For COParea, the treatment effect for the difference between pooled pretest and posttest means for the CCT group approached a medium effect, indicating a detectable improvement in postural stability at posttest. Effect sizes associated with SR stimulation ranged from medium to high, indicating postural stability improved at posttest. Cohen [33] defines low, medium, and high effect sizes as 0.30, 0.50, and 0.80, respectively.

**Table 2:** Treatment Effects Associated With Posttest Improvements In Postural Stability Compared To The Pretest Pooled Means.

	Control	CCT	Pooled (Control + CCT)	SCT
AP COPvel	0.18	0.17	0.18	0.87
M/L COPvel	0.13	0.27	0.21	0.71
M/L COPsd	-0.77	0.11	-0.34	0.77
M/L COPmax	-0.15	-0.08	-0.10	0.45
COParea	0.12	0.37	0.25	0.63

Effect size values are present for the control group, conventional coordination training (CCT) group, pooled posttest mean of the control and CCT groups, and the stochastic resonance stimulation coordination training (SCT) group for the following measures: Anterior/Posterior Center-of-Pressure Velocity (A/P COPvel); Medial/Lateral Center-of-Pressure Velocity (M/L COPvel); Medial/Lateral Center-of-Pressure Standard Deviation (M/L COPsd); Center-of-Pressure Area (COParea); and Medial/Lateral Center-of-Pressure Maximum Excursion (M/L COPmax). Positive effect size values indicate posttest postural stability improvements. Negative effect size values indicate posttest postural stability impairments.

## Discussion

The most important findings of this study indicate that SR stimulation used as an adjunct therapy to coordination training enhanced postural stability deficits associated with FAI. Subjects participating in six weeks of coordination training with SR stimulation had better postural stability than subjects training without SR stimulation and control subjects at posttest. Furthermore, treatment effects associated with SR stimulation were greater than effects associated with coordination training alone. Of particular importance were improvements in COPvel and M/L COPsd following training with SR stimulation. Faster COPvel and greater M/L COPsd have been indicative of ankle sprain injury in the physically active [15,16]. Thus, SR stimulation has implications for treating and preventing ankle sprain injury associated with FAI since this stimulation slowed COPvel and reduced M/L COPsd.

Single leg stance postural stability has also improved with SR stimulation applied to the lower extremity of healthy subjects, elderly, and diabetic patients [27-30]. Furthermore, SR stimulation applied during single leg balance has improved postural stability (COPvel) in subjects with FAI when compared to single leg balance without SR stimulation [34]. Our current results indicate that postural stability as measured by COP measures (COPvel, COPsd, COPmax, COParea) can be enhanced following six weeks of coordination training with SR stimulation after the stimulation was removed. These results have clinical significance, as clinicians can rehabilitate individuals with FAI using SR stimulation for several weeks, and then return individuals to full physical activity with enhanced postural stability.

Potential mechanism whereby SR stimulation improved postural stability in this current investigation might be related to improvement in signal detection and enhancement of motor system function. Stochastic resonance stimulation has been reported to act directly on muscle spindle mechanoreceptors or indirectly through cutaneous fusimotor reflexes to enhance signal detection [31]. Enhanced detection of signals related to postural control could have improved postural stability in the SCT group. In addition to affecting the sensory system, SR stimulation has been reported to affect the motor system in the muscle spindle motoneuron synapse by modulating monosynaptic reflexes generated from muscle spindles [32]. This type of SR phenomenon has potential for improving sensorimotor deficits associated with FAI. Arthrogenic muscle inhibition is a sensorimotor deficit associated with FAI, and has been implicated as a causal factor of FAI, as depressed maximal H-reflex to maximal M-wave (H:M) ratios have been associated with FAI [35]. A therapy such as SR stimulation eliciting greater monosynaptic reflexes has implications for improving arthrogenic muscle inhibition by facilitating muscle activation. Thus, greater dynamic ankle joint stability may result from SR stimulation. In our current study, six weeks of coordination training with SR stimulation might have introduced neuroplastic changes that increased muscle activation, thereby improving postural stability.

The results of this current investigation are similar to results reported in other coordination training investigations [21,22]. Wobble board training with strips of athletic tape applied to the lateral aspect of the foot and ankle of subjects with FAI has improved single leg postural stability (COParea) more than wobble board training without tape after six weeks of training [21]. Proprioception might have improved by athletic tape stimulating cutaneous receptors during wobble board training [21]. In a related investigation to our current study, the effects of SR stimulation on dynamic postural stability (time-to-stabilization) were examined, and the results indicated that coordination training with SR stimulation might enhance dynamic postural stability in subjects with FAI earlier and to a greater extent than coordination training alone after four weeks of training [22].

Coordination training alone has improved postural stability in subjects with FAI [17-22]. The medium treatment effect (0.37) associated with CCT group's COParea suggests that postural stability improved COParea following coordination training. This medium treatment effect, however, was not as high as the treatment effect (0.63) associated the SCT group's COParea. This higher effect in the SCT group suggests that coordination training with SR stimulation facilitates rehabilitation more than coordination training alone.

Researchers have also reported that coordination training alone has not impacted certain single leg balance COP measures of subjects with FAI [18,20,25]. These results concur with our current findings, as the CCT group did not enhance subjects' postural stability to a greater extent than the control group. Additionally, the moderately negative treatment effect associated with the M/L COPsd in the control group indicates that postural stability worsened at posttest. We do not know the reason for this negative treatment effect. Negative treatment effect for the control group indicates that the M/L COPsd was not a valid or reliable measure of postural stability in this study.

Our orthogonal contrast provided a statistical technique to detect a treatment effect of SR stimulation on postural stability. The rationale for using orthogonal contrasts were based on results presented by several researchers, who reported that learning effects were responsible for COP excursion improvements in both balance training and control subjects [18-20]. Additionally, Verhagen et al [36] did not find group posttest differences between training and control groups. Thus, we believed that differences might not occur between control and CCT group posttest means in this current investigation. The first orthogonal contrast comparing control and CCT groups in our study was established based on this speculation. The second orthogonal contrast examined the effects of SR stimulation compared to the pooled posttest means of control and CCT groups. Our results indicate that coordination training alone did not result in significantly better postural stability than subjects who did not participate in coordination training at posttest. Since differences were not evident, the pooled means of the control and CCT groups were then compared to the SCT group's means to detect treatment effects associated with SR stimulation. Thus, our results indicate that SR stimulation might be used as an alternative therapy to improve postural stability deficits associated with FAI.

Coordination training that enhances postural stability has implications in preventing ankle sprain injury [1,20,24]. Alternative therapies that improve postural stability to a greater extent than coordination training alone might also help prevent ankle sprain injury. Coordination training with SR stimulation is one such alternative therapy that can be used clinically to improve postural instabilities associated with FAI. Future research should confirm our findings with a larger sample size and should examine the effects SR stimulation has on the prevention of recurrent ankle sprain injury in physically active individuals with FAI.

## Abbreviations

A/P: Anterior/Posterior;

CCT: Conventional Coordination Training;

COP: Center-of-Pressure;

COParea: Center-of-Pressure Area;

COPmax: Center-of-Pressure Maximum Excursion;

COPsd: Center-of-Pressure Standard Deviation;

COPvel: Center-of-Pressure Velocity;

FAI: Functional Ankle Instability;

M/L: Medial/Lateral;

SR: Stochastic Resonance;

SCT: Stochastic Resonance Stimulation Coordination Training.

## Competing interests

The author(s) declare that they have no competing interests.

## Authors' contributions

All authors contributed to the conception and design of this study, and the analysis and interpretation of data. SER, JTB, and CNB were involved in the acquisition of data. All authors have been involved in drafting the manuscript, and revising it critically for important intellectual content. All authors have given approval of the final version.
